# CO_2_ Loss into Solution: An Experimental Investigation of CO_2_ Electrolysis with a Membrane Electrode Assembly Cell

**DOI:** 10.1021/acsaem.4c01101

**Published:** 2024-09-05

**Authors:** Weiming Liu, Harry Dunne, Bernardo Ballotta, Allyssa A. Massie, Mohammad Reza Ghaani, Kim McKelvey, Stephen Dooley

**Affiliations:** † School of Physics, Trinity College Dublin, Dublin D02 PN40, Ireland; ‡ School of Engineering, Department of Civil, Structural & Environmental Engineering, Trinity College Dublin, Dublin D02 PN40, Ireland; § MacDiarmid Institute for Advanced Materials and Nanotechnology, School of Chemical and Physical Sciences, Victoria University of Wellington, Wellington 6140, New Zealand

**Keywords:** membrane electrode assembly cell, CO_2_–OH^−^ neutralization, pure silver membrane cathode, gas diffusion electrode, electrochemical CO_2_ reduction reaction (ECO_2_RR), hydrogen evolution reaction (HER)

## Abstract

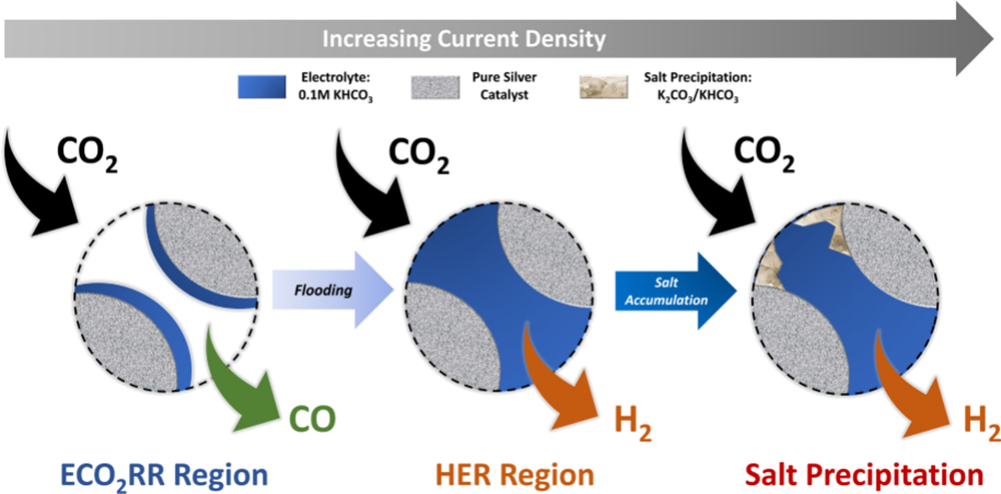

In pursuit of commercial viability for carbon dioxide (CO_2_) electrolysis, this study investigates the operational challenges associated with membrane electrode assembly (MEA)-type CO_2_ electrolyzers, with a focus on CO_2_ loss into the solution phase through bicarbonate (HCO_3_^–^) and carbonate (CO_3_^2–^) ion formation. Utilizing a silver electrode known for selectively facilitating CO_2_ to CO conversion, the molar production of CO_2_, CO, and H_2_ is measured across a range of current densities from 0 to 600 mA/cm^2^, while maintaining a constant CO_2_ inlet flow rate of 58 mL/min. The dynamics of CO_2_ loss are monitored through measurements of pH changes in the electrolyte and carbon elemental balance analysis. Employing the concept of conservation of elemental carbon, a chemical reaction analysis is conducted, identifying the critical role of the hydroxide (OH^–^) ion. At lower current densities below 125 mA/cm^2^, where CO_2_ reduction predominates, it is observed that CO_2_ loss is proportional to current density, reaching up to 0.18 mmol/min, and directly correlates with the rate of OH^–^ ion production, indicative of HCO_3_^–^/CO_3_^2–^ ion formation. Conversely, at higher current densities above 450 mA/cm^2^, where hydrogen evolution is the dominant process, CO_2_ loss is shown to decouple from the OH^–^ ion production rate with a constant limit condition of 0.12 mmol/min, regardless of the current density. This suggests that electrolyte-induced cathode flooding restricts CO_2_ access to cathode sites. Additionally, pH change in the electrolyte during the electrolysis further infers differing ion populations in the CO_2_ reduction and hydrogen evolution regimes, and their movement across the membrane. Continued monitoring of the pH change after the cessation of electricity offers insights into the accumulation of HCO_3_^–^/CO_3_^2–^ ion at the cathode, influencing salt formation.

## 1 Introduction

Electrochemical carbon dioxide reduction has emerged as a key innovation for transitioning the global energy framework toward net-zero emissions.^1^ Converting CO_2_ into valuable outputs such as fuel precursors can significantly reduce dependence on fossil fuels. These outputs, acting as renewable hydrocarbons, not only encourage CO_2_ capture by transforming it into useful products but also offer a strategic approach to storing excess renewable energy in hydrocarbon form.^2^ This storage capability facilitates the subsequent utilization of this energy, further advancing sustainability goals.

However, traditional liquid-phase CO_2_ electrolyzers encounter mass transport limitations due to the low solubility of CO_2_ (33 mM)^3^ and its slow diffusivity (1.9 × 10^–5^ cm^2^/s)^4^ in the aqueous solution, which constrains the current density of electrochemical CO_2_ reduction reaction (ECO_2_RR) within 20 mA/cm^2^.^5^ This is well below the minimum industrial requirement of at least 200 mA/cm^2^,^6^ impeding its commercial application. Responding to these challenges, CO_2_ electrolyzer designs have advanced with the integration of a gas diffusion layer (GDL), a critical development that enhances mass transport, facilitating gas-phase delivery of CO_2_ to the reaction sites on the electrode.^7,8^ This innovation typically achieves a current density an order of magnitude higher than that possible with a liquid-phase system,^9^ representing a significant step forward in overcoming the barriers to the commercial viability of ECO_2_RR.

In the transition to gas-diffused electrolyzers for CO_2_ electrochemical reduction, the membrane electrode assembly (MEA) technology stands out,^10^ which requires precisely layering the cathode and anode gas diffusion electrodes against an anion exchange membrane (AEM). This MEA configuration, by eliminating the catholyte stream, significantly reduces ohmic losses and thereby lowers the operating voltage,^11^ demonstrating substantial potential for commercial success. However, even with these advantageous characteristics for scaling up, the technology faces ongoing challenges, particularly with CO_2_ utilization efficiency and the durability of the cells.

Previous research has demonstrated that the efficiency of single-pass CO_2_ utilization in MEA-type cells is capped at approximately 50%, a limitation highlighted by both simulations^12−16^ and experimental studies.^15,17,18^ The results from the hydroxide ions produced during both ECO_2_RR and the hydrogen evolution reaction (HER) within the cell’s operational environment are crucial, as hydroxide ions consume a considerable amount of the incoming CO_2_ via CO_2_–OH^–^ neutralization reactions (CO_2(aq)_ + OH^–^_(aq)_ ⇋ HCO_3_^–^_(aq)_ + OH^–^_(aq)_ ⇋ CO_3_^2–^_(aq)_ + H_2_O_(aq)_).^19^ Moreover, the resulting (bi)carbonate ions combine with cations, typically K^+^_(aq)_, within the cell, leading to salt precipitation.^20^ This will clog the cathode’s porous structure and drastically reduce the cell’s operational lifespan, sometimes to just a few hours, a challenge that is well-documented across numerous studies.^21−24^

Herein, this study aims to elucidate the process of CO_2_ loss to the solution phase by separately tracking OH^–^ ion formation through ECO_2_RR and HER under a CO_2_-rich environment. This analysis clarifies how each contributes to the formation of bicarbonate and carbonate ions, which are central to understanding the dynamics of CO_2_ transport and loss.

To this end, the elemental carbon balance was quantified in the cathodic compartment of an MEA-AEM cell featuring a porous silver electrode. The experimental observations suggest a hypothesis that the formation of (bi)carbonate ions represents the principal competitive process in CO_2_ electrochemical reduction at lower current densities. Furthermore, at elevated current densities, CO_2_ loss reaches a plateau during HER, a phenomenon indicative of electrolyte-induced cathode flooding occurring. This flooding results in a fraction of the pores within the gas diffusion electrode being filled by liquid, thereby confining the supply of CO_2_ to the reaction sites. Additionally, through the continuous monitoring of the electrolyte’s pH changes, coupled with carbon balance analysis, it has been revealed that flooding combined with HER accelerates the precipitation of (bi)carbonate salts at the cathode.

Building on these insights, the following sections describe the experimental approach designed to explore the formation of (bi)carbonate ions under different conditions and the effects of cathode flooding on salt precipitation. By elucidating these mechanisms, this research could contribute to the development of more durable and efficient ECO_2_RR systems.

## 2 Experimental Section

### 2.1 Materials and Cell Assembly

**Figure 1:**
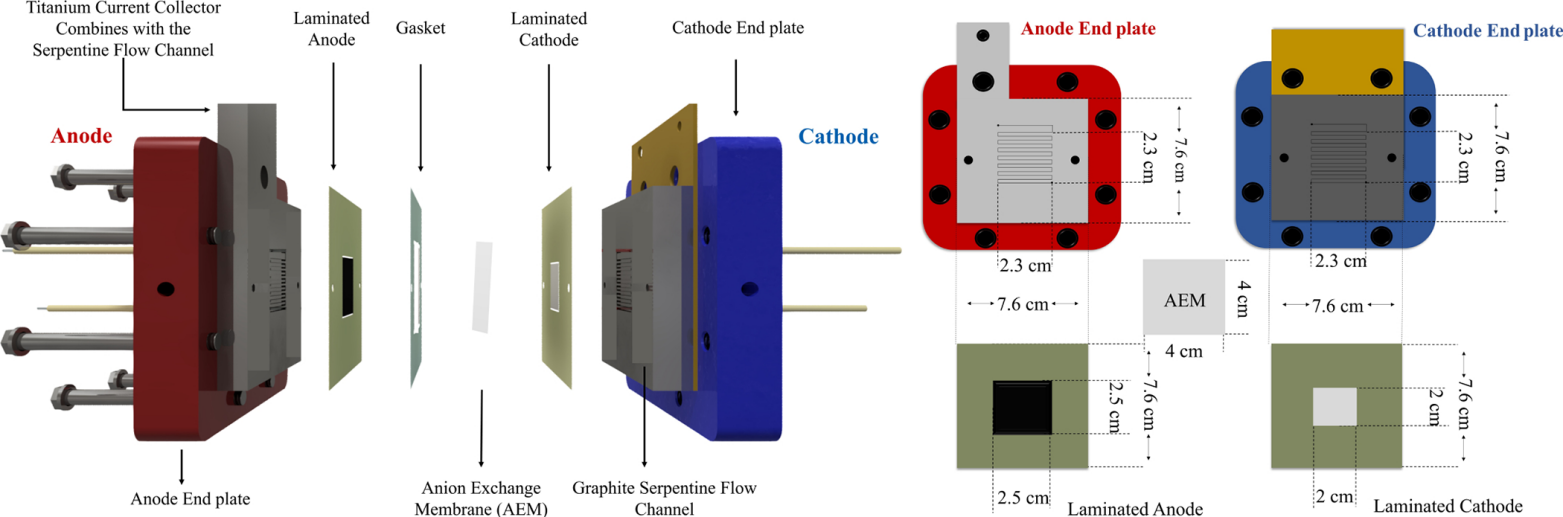
Schematic of the membrane assembly electrode-anion exchange membrane electrolyzer for CO_2_ reduction with a gas-fed cathode and a liquid-fed anode, the geometrical parameters of major components are presented.

A commercially available electrolyzer sourced from Scribner Associates is employed in this study. This electrolyzer features a titanium current collector for the liquid-fed anode end plate and a gold-plated copper current collector for the gas-fed cathode end plate, both with serpentine flow channels, as illustrated in Figure 1. For the cell’s cathode, a 50 μm thick silver filtration membrane with pore sizes of 0.2 μm (active area of 4 cm^2^), serving as the gas diffusion electrode, is obtained from Pieper Filter GmbH (SEM images in Figure S1). Silver as a catalyst favors CO production in ECO_2_RR^25,26^ and the pure silver membrane utilized in this investigation offers multifunctional advantages, including catalytic activity, mechanical support, and current collection capabilities.^27^ Additionally, this type of silver cathode avoids the problem of the carbon supports degrading in alkaline media, which would shorten the cell lifespan.^22^ The anode is a 370 μm thick IrO_2_/Carbon fiber composite (SEM images in Figure S2) purchased from Dioxide Materials. Two 75 μm thick polyethylene terephthalate (PET) laminator sheets are used to encase and align each electrode. A Sustainion X37–50 grade 60 anion-exchange membrane, procured from Dioxide Materials, is incorporated into the cell assembly as the ionic exchange membrane, selected specifically for its low electrical resistance and high selectivity.^28,29^ Ideally, this membrane supports exclusive anion transport with minimal proton permeability and exhibits high OH^–^ conductivity—qualities that are essential for preventing H^+^ migration and accumulation at the cathode and thus effectively suppressing the hydrogen evolution reaction (HER).^28^ To mitigate risks associated with prolonged high pH exposure and potential membrane degradation, our experiments are conducted over short durations—only a couple of hours each to minimize the impact of membrane degradation. Before cell assembly, the AEM is immersed in 1 M KOH over 24 h for activation and rinsed with deionized water before the assembly.

In the assembly process, components are layered in sequence as depicted in Figure 1. To guarantee gas and water tightness, the complete cell assembly is secured with a torque of 11 Nm.

### 2.2 Reaction and Measurement System

**Figure 2:**
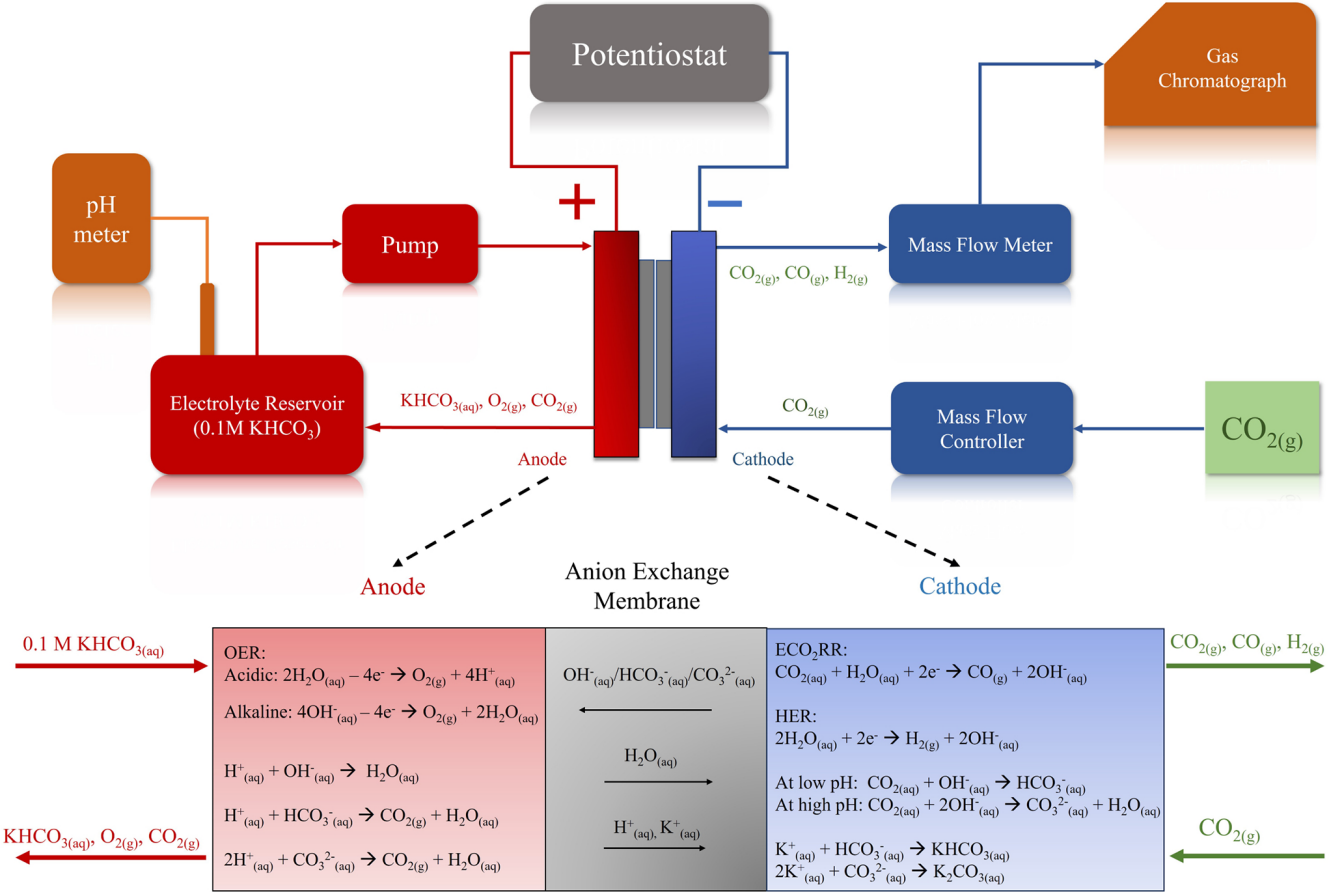
Measurement system of the membrane electrode assembly—anion exchange membrane electrochemical cell for CO_2_ reduction and an illustration of the possible reactions occurring on the electrodes.

The measurement system has been established and summarized in Figure 2. On the cathode side, nonhumidified CP-grade CO_2_ (≥99.8% purity) is introduced into the cathode inlet of the reactor at a controlled rate of 58 mL/min (2.31 mmol/min), regulated by a mass flow controller (Bronkhorst model: FG-201CV-AGD-33-K-DA-000). Unreacted CO_2_ and potential gaseous products exit through the cathode outlet, being monitored by a mass flow meter (ALICAT Mass Flow Meter M, 0–500SCCM). Manual control valves enable gas component analysis with an Agilent 8860 Gas Chromatograph with a column configuration of 6ft1/8-in. Porapack Q and 6ft1/8-in. Molecule Sieve in series and using helium as the carrier gas.

On the anode side of a 3 L electrolyte, 0.1 M KHCO_3_, is prepared by dissolving 30.033 g potassium hydrogen carbonate (KHCO_3_, ≥99% purity, from Fisher Scientific) in 3 L deionized water (SimPak 2 Purification Cartridge, Resistance: 18 MΩ). The electrolyte is circulated to and from the reactor using a peristaltic pump (MasterFlex 07528–10) at a flow rate of 8.2 mL/min. A portable meter (Multi 3630 IDS Xylem-WTW 2FD570), coupled with a pH sensor is employed to monitor the pH variations in the electrolyte constantly throughout the experiments.

For the implementation of the electrochemical technique, input potential or current for reactions is precisely controlled using an Ivium Vertex potentiostat (5 A, ±5 A, ±10 V). All experiments reported in this study are conducted under ambient conditions (20–25 °C, around 101 kPa).

The reproducibility of the experiments is thoroughly evaluated by conducting three separate trials, each with a newly fabricated cell, under identical conditions, and subsequently comparing the concentrations of CO produced in each trial. Two sets of inlet flow rates of CO_2_ are tested (58 and 147 mL/min). The standard deviation remains below 0.03, with a coefficient of variance around 0.1 in all tested cases, as illustrated in Figure S3.

#### 2.2.1 Reactions on the Catalyst and the Carbon Cross-Over Phenomenon

With this robust experimental setup established, pure metallic silver and IrO_2_/Carbon materials are utilized for the cathode and anode, respectively, where the global electrochemical reactions are currently understood to be depicted in Figure 2,

Electrochemical reactions on the cathode
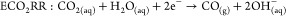
1
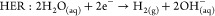
2

Electrochemical reactions (Oxygen evolution reaction) on the anode
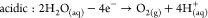
3
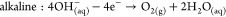
4

In the context of CO_2_ reduction, HER is a parasitic reaction, competing with ECO_2_RR, particularly at high applied currents, where HER prevails.^10,29−32^ Additionally, both reactions on the cathode make the local environment alkaline due to the OH^–^ formation, which proposed a mechanism that results in a phenomenon of CO_2_ loss into the solution phase by the assumed nonelectrochemical reactions below^19,33^
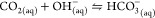
5
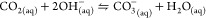
6

With an electrical field applied to the reactor, HCO_3_^–^ and CO_3_^2–^ migrate from the cathode, across the AEM to the anode, and release gaseous CO_2_ by being neutralized with protons generated on the anode, depicted in the following reactions^34^
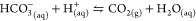
7
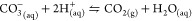
8

Reactions 9 and 10 describe the risk of salt buildup on the cathode. This occurs as the cathode’s vicinity accumulates high concentrations of HCO_3_^–^ and CO_3_^2–^, which then react with freely moving K^+^ ions to form KHCO_3_ and K_2_CO_3_ as the reactions below
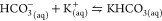
9
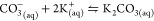
10

Once the concentrations of potassium (bi)carbonate reach the solubility limit (7.93 M K_2_CO_3_ and 2.24 M KHCO_3_ at 20 °C in pure water^4^) in the local environment, these salts will precipitate and obstruct the cathode’s porous structure, degrading the efficiency and durability of the cell.^23,35^ This process is identified as one of the main challenges in ECO_2_RR. Understanding the CO_2_ “cross-over” phenomenon is thus crucial. By quantitatively assessing the elemental carbon balance, this study aims to elucidate the mechanisms behind CO_2_ loss to the solution phase, offering valuable insights into one of ECO_2_RR’s primary challenges.

#### 2.2.2 Quantification of the Elemental Carbon Balance

Quantification of the elemental carbon balance is essential for accurately assessing CO_2_ loss into the solution phase during electrochemical reactions. It necessitates precise measurements of two critical parameters: the initial CO_2_ concentration entering the system (CO_2_ inlet) and the total volume of gases emitted from the cell, including both unreacted CO_2_ and CO generated through the ECO_2_RR, each with its specific molar flow rate. Accordingly, the carbon elemental balance in molar per second (mol/s) within the cathodic compartment is represented by the equation
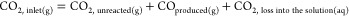
11

To compute the production rate of each species, the product concentrations are multiplied by the volume flow rate. The molar quantities of these products are then calculated using the ideal gas law
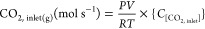
12
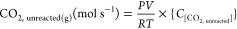
13
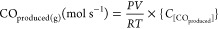
14
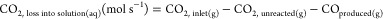
15where, the *V* presents the volume flow rate of the effluent gas, the concentrations (*C*) of the respective gases are determined via gas chromatography. All the experiments were conducted under standard ambient conditions. Therefore, *P*, *T* and *R* (gas constant) are 101,350 Pa, 300 K and 8.314 J/(mol × K), respectively.

#### 2.2.3 Faraday Efficiency Calculation

For precise selectivity analysis of electrochemical reactions, the Faraday efficiency (F.E.) can be calculated using eq 16. This calculation incorporates the molar quantities determined earlier, enabling accurate analysis of the elemental carbon balance in the cathodic compartment.
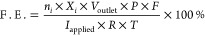
16Where, *n*_*i*_ represents the number of electrons transferred to the species *i*, *X*_*i*_ denotes the molar concentration of species *i* within the effluent gas, *V*_outlet_ is the flow rate of the effluent gaseous products, also *I*_applied_ and *F*, corresponds to the applied electrical current and the Faraday constant (96485.332 C/mol) respectively.

## 3 Results and Discussion

The theoretical framework discussed in Section 2.2.1 suggests that the loss of CO_2_ into the solution primarily occurs through mechanisms driven by OH^–^ generation, as detailed in Reactions 5 and 6. This assumption highlights the need for experimental verification of the link between OH^–^ generation and CO_2_ loss.

Directly measuring the concentration of OH^–^ ions within the cell presents significant technical challenges. Nevertheless, the generation of CO and H_2_ during electrochemical processes can serve as an indirect indicator of OH^–^ production according to Reactions 1 and 2, where the stoichiometric ratios of CO and H_2_ production relative to OH^–^ are both established at 1:2. Therefore, by quantifying CO and H_2_ outputs, the OH^–^ ion generation can be indirectly assessed.

Moreover, the distinction between OH^–^ production from the ECO_2_RR and HER is fundamental for understanding the dynamics of CO_2_ loss. This insight is crucial for optimizing electrochemical conditions to minimize CO_2_ loss and limit unwanted hydrogen production. To track and differentiate OH^–^ ion production by ECO_2_RR and HER in a CO_2_-rich environment, Haspel and Gascon^36^ utilized distinct electrode materials designed to selectively facilitate ECO_2_RR while others favor HER. However, employing various electrode materials might introduce additional variables, complicating the interpretation of experimental results.

In this study, the effects of HER and ECO_2_RR are adjusted solely through adjustments in the applied current density while maintaining all other operational parameters constant. The observation reveals that the ECO_2_RR is more pronounced at lower current densities, and HER becomes predominant at relatively higher currents.

**Figure 3:**
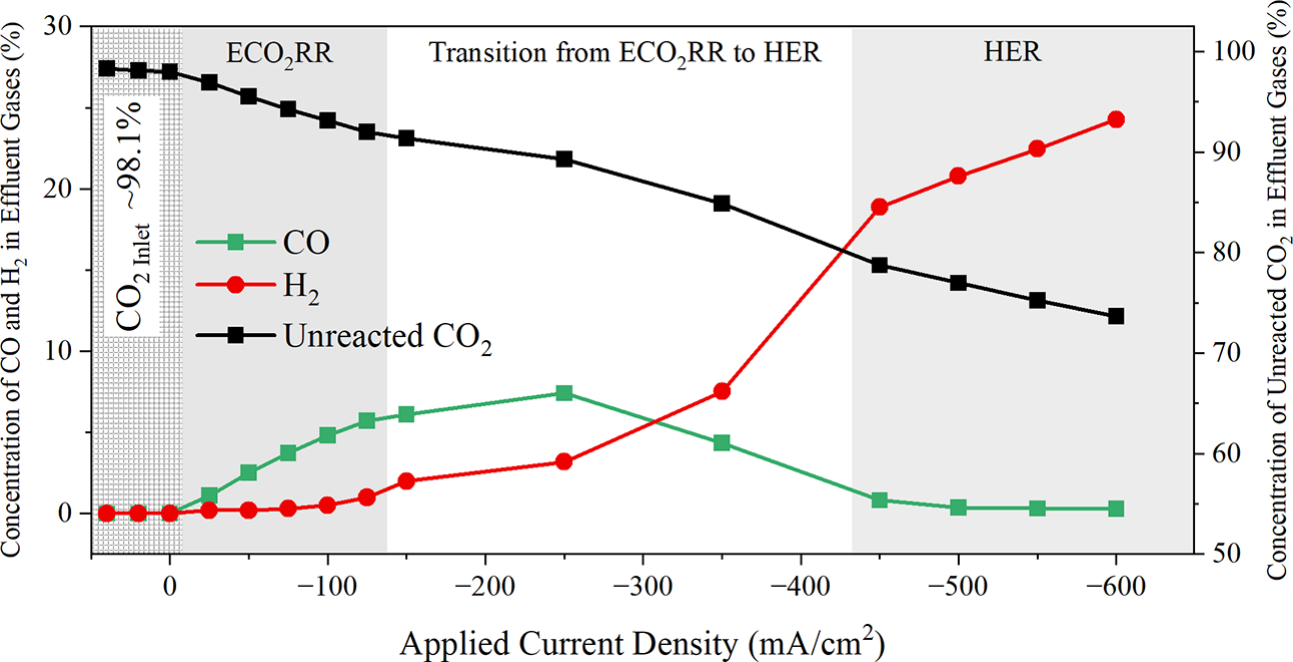
Mole concentration of H_2_, CO and CO_2_ in the effluent gas as a function of the applied current density from 0 to 600 mA/cm^2^. The initial CO_2_ concentration at the inlet is determined at 98.1% and serves as the baseline, as indicated in the figure. Experimental conditions include a 0.1 M KHCO_3_ electrolyte with a circulating flow rate of 8.2 mL/min and a constant inlet CO_2_ flow rate of 58 mL/min under ambient conditions (∼25 °C ± 2).

To define the specific operational regimes of ECO_2_RR and HER within this electrochemical framework, a detailed examination was conducted using chronopotentiometry (CP) across a board current density range from 25 to 600 mA/cm^2^ (illustrated in Figure S4), coupled with the analysis of gaseous product concentrations to capture the variances in CO and H_2_ production in relation to applied current density shifts, as depicted in Figure 3. The ECO2RR region, identified at applied current densities below 125 mA/cm^2^, is marked by predominant CO production with negligible H_2_ generation (less than 0.5%). Conversely, the HER region, effective at current densities over 450 mA/cm^2^, features increasing H_2_ production linearly, while CO generation is maintained below 0.5%. The area between these current densities represents a transitional phase, in which the system goes from being dominated exclusively by ECO_2_RR to the progressive activation of HER, indicating a combination and mutual influence of both reactions which affects the overall concentrations of the chemical species involved.

As outlined earlier, our study hypothesizes that CO_2_ loss into the solution is proportionate to OH^–^ formation across both the ECO_2_RR and HER regimes, as detailed in Reactions 5 and 6. This proportional relationship is further expected to correlate with the production of CO and H_2_, given that the same amount of OH^–^ is generated from both ECO_2_RR and HER, according to Reactions 1 and 2.

To systematically test this hypothesis, the study is structured to explore the ECO_2_RR domain (currents from 25 to 125 mA/cm^2^) and the HER domain (currents from 450 to 600 mA/cm^2^) in separate sections, aiming to precisely track the OH^–^ generation from both reactions individually as well as elucidate the distinct behaviors and impacts of these reactions on CO_2_ loss into the solution phase.

### 3.1 ECO_2_RR Region

**Figure 4:**
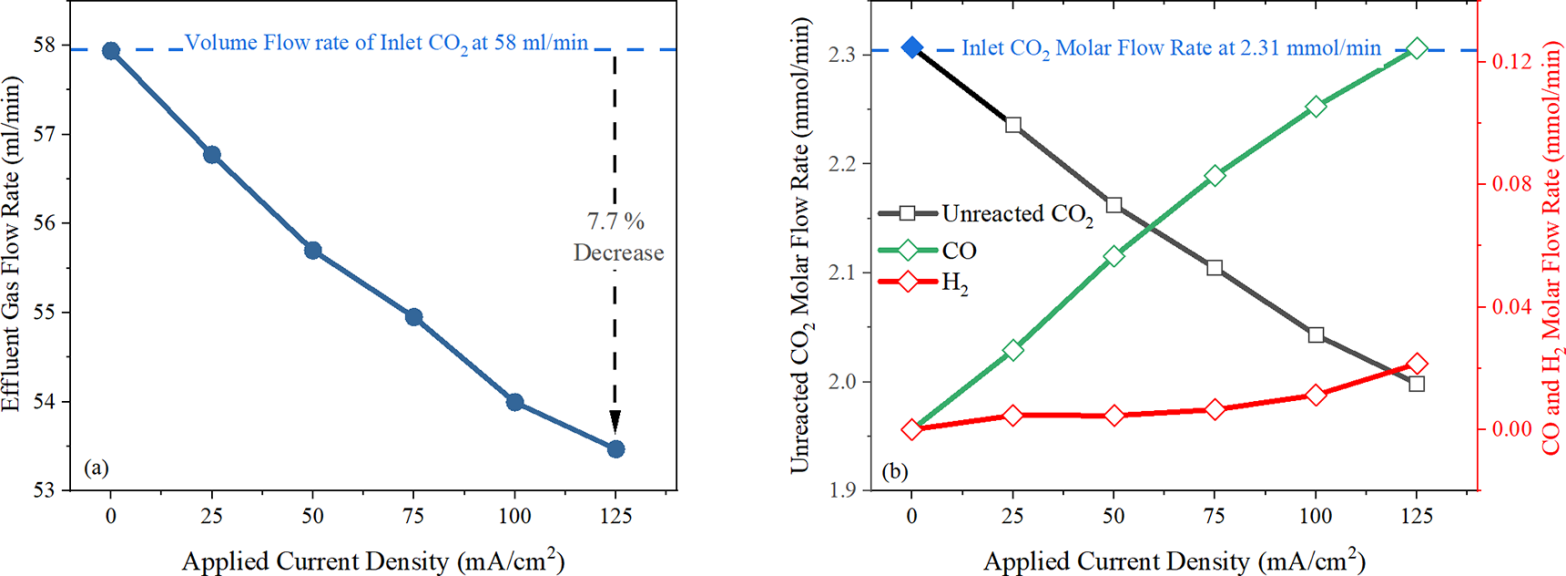
(a) Illustrates the volume flow rate of effluent gas recorded from the cell corresponding to the increasing applied current densities. The top dashed line presents the volume flow rate of CO_2_ entering the system. (b) Shows the calculated molar flow rate of each effluent gaseous product composition varies along with the increasing applied current densities. The top dashed line indicates the molar flow rate of CO_2_ fed into the system. Experimental conditions include a 0.1 M KHCO_3_ electrolyte with a circulating flow rate of 8.2 mL/min and a constant inlet CO_2_ flow rate of 58 mL/min under ambient conditions (∼25 °C ± 2).

Following the discussion above, the experimental outcomes for the ECO_2_RR region are presented in Figure 4 to demonstrate its unique contributions to CO_2_ loss.

Theoretically, each mole of CO_2_ consumed should yield an equivalent mole of CO gas regarding Reaction 1. Consequently, in the absence of substantial H_2_ production, the volume flow rates of inlet CO_2_ and the existing gas should be the same. However, at higher current densities under the ECO_2_RR region, a noticeable decrease of 7.7% in the effluent volume relative to the total is observed, as shown in Figure 4a, which challenges the anticipated reaction stoichiometry but supports the hypothesis that CO_2_ might be alternatively lost through absorption into the solution phase.

Additionally, the molar flow rate of effluent gases, calculated according to the equations presented in Section 2.2.2 (eqs 12–15), illustrated in Figure 4b, where the reduction of CO_2_ is 0.31 mmol/min and the amount of CO produced reaches up to 0.125 mmol/min at elevated applied current densities in the ECO_2_RR region. This indicates that more than half of the consumed CO_2_ does not contribute to the observed gaseous products, also suggesting its loss into the solution phase.

Moreover, the combined Faraday efficiency of H_2_ and CO production, nearly 100% as detailed in Figure S5, highlights the system’s selectivity toward these products. This high-efficiency level minimizes the likelihood of alternative electrochemical pathways, thus emphasizing the primary conversion of CO_2_ to CO as well as the loss mechanism of CO_2_ into the solution through nonelectrochemical processes.

**Figure 5:**
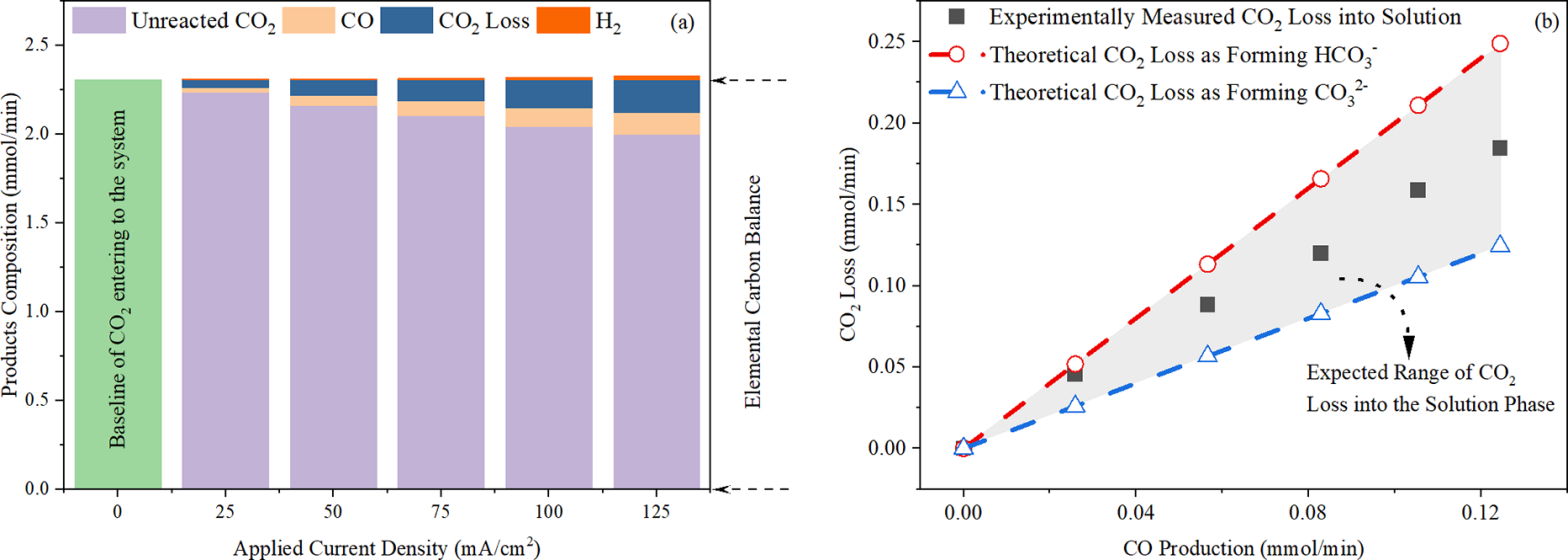
(a) Molar flow rate of the composition of each product plotted against applied current density in the ECO_2_RR region. Elemental carbon balance marked includes the unreacted CO_2_, CO produced and the CO_2_ loss into the solution phase. (b) Molar flow rate of CO_2_ loss plotted as a function of CO production rate, derived from the data presented in Figure 5a. The black dots represent experimental CO_2_ loss against CO production; the red dashed line indicates the theoretical CO_2_ loss against CO production with the ratio of 2:1, considering bicarbonate formation; the blue dashed line presents the theoretical CO_2_ loss against CO production with the ratio of 1:1, considering carbonate formation. Experimental conditions include a 0.1 M KHCO_3_ electrolyte with a circulating flow rate of 8.2 mL/min and a constant inlet CO_2_ flow rate of 58 mL/min under ambient conditions (∼25 °C ± 2).

To confirm our hypothesis that the CO_2_ lost into the solution phase is due to the nonelectrochemical processes of forming (bi)carbonate ions, further data analysis was conducted to evaluate the correlation between CO_2_ loss and CO production. The elemental carbon balance on the cathodic compartment of the cell is calculated with eq 11 and illustrated in Figure 5a, where the proportion of CO_2_ loss in the solution exceeds the H_2_ production across the applied current densities. This trend is responsible for the decrease in the volume flow rate of the effluent gaseous products. Also, Figure 5b demonstrates a direct correlation between increased CO production and the loss of CO_2_ to the solution. It supports our theoretical framework that the enhanced CO production leads to an increment in OH^–^ formation, which in turn accelerates the loss of CO_2_. This process primarily leads to the transformation of CO_2_ into bicarbonate (HCO_3_^–^) and carbonate (CO_3_^2–^) ions. This is achieved through the net reactions resulting from the combination of Reaction 1 with the solution phase reaction (Reaction 5) for bicarbonate, and Reaction 6 for carbonate, respectively.
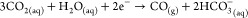
17

This reaction presents the 1:2 ratio of CO production to CO_2_ loss, where for every molecule of CO produced, two molecules of CO_2_ react with OH^–^ produced during the electrochemical reduction to form bicarbonate.
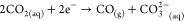
18

This pathway reflects a 1:1 ratio of CO production to CO_2_ loss, representing the direct conversion of CO_2_ to carbonate in the presence of excess OH^–^.

The alignment of experimental data with theoretical predictions is clearly demonstrated, with all data points falling within the expected ranges between the red and blue dashed lines. The shaded area indicates the transition zone where both bicarbonate and carbonate ions are formed.

Furthermore, the findings indicate that a lower rate of CO production primarily leads to the formation of HCO_3_^–^, whereas at a higher rate of CO production, CO_3_^2–^ become increasingly dominant. This shift is consistent with observations made in other experimental studies,^36−38^ which established that carbonate ions are the primary species migrating through the membrane, indicating a preference for CO_3_^2–^ conversion at higher current densities. This shift from bicarbonate to carbonate ion predominance can be attributed to the increase in OH^–^ ion generation that accompanies higher rates of CO production.

In the experiments, a 0.1 M KHCO_3_ solution, with an initial pH of 8.4, was circulated within the cell as the electrolyte before any experimental measurements were taken, ensuring a uniform initial pH level throughout the cell. As the applied current density is increased, the resulting rise in OH^–^ ion concentration makes the local environment at the cathode more alkaline, shifting the chemical equilibrium toward carbonate ion formation, described as the reaction below^24^
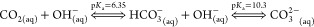


These shifts occur at pH levels corresponding to p*K*_a_ values of 6.35 for the first reaction and 10.3 for the second, indicating the preferential formation of carbonate ions at higher pH levels typical of increased OH^–^ production.

Additionally, Marcandalli et al.,^39^ suggest another role for bicarbonate: as a beneficial proton donor that enhances CO production in the ECO_2_RR region, providing an alternative mechanism to the direct transition from HCO_3_^–^ to CO_3_^2–^.

Those observations from the ECO_2_RR region substantiate our theoretical model, highlighting OH^–^ generation as a key factor in CO_2_ loss. This suggests that the formation of (bi)carbonate ions represents a significant competitive pathway in the electrochemical reduction of CO_2_, particularly in the ECO_2_RR region at lower applied current densities.

### 3.2 HER Region

Exploration of the HER region uncovers a unique mechanism for CO_2_ loss, different from the mechanism observed in the ECO_2_RR region. This distinction highlights the differing processes of CO_2_ mass transport to the reaction sites across the ECO_2_RR and HER domains.

According to Reactions 1 and 2, both the HER and ECO_2_RR generate an equivalent amount of OH^–^ ions per unit of electrical input. Considering the significant role of OH^–^ in CO_2_ loss into the solution, it would be straightforward to assume that under the HER conditions, increased OH^–^ ions concentration converts inlet CO_2_ to HCO_3_^–^ and CO_3_^2–^, mirroring the CO_2_ loss mechanism observed in the ECO_2_RR region. The net reactions, combining the electrochemical reaction with solution phase reactions that contribute to CO_2_ loss in the HER scenario, are depicted below
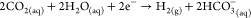
19
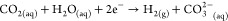
20

**Figure 6:**
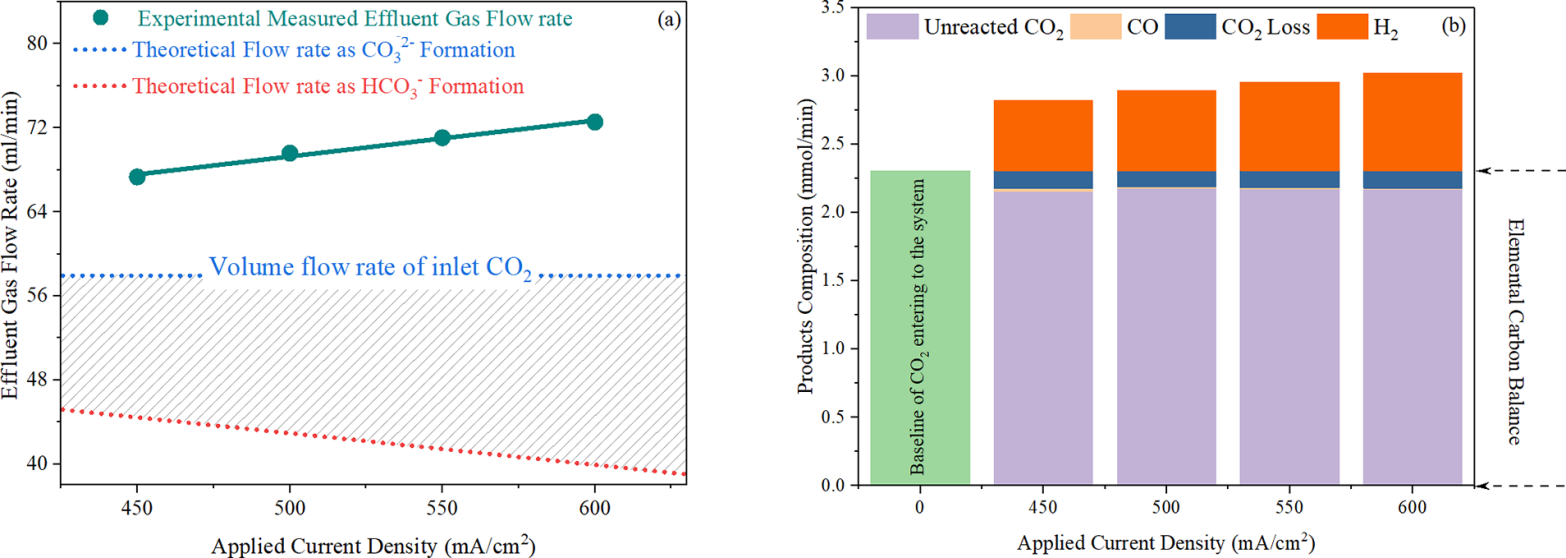
(a) Illustrates the volume flow rate of the effluent gas from the cell corresponding to the increasing applied current densities in the HER region with the applied current density above 450 mA/cm^2^. The blue dashed line indicates the volume flow rate of CO_2_ fed into the system, equal to the anticipated volume flow rate considering carbonate ions formation. The red dashed line presents the theoretical volume flow rate of effluent gas, considering bicarbonate ions formation. (b) Shows the calculated molar flow rate of each product composition coming out of the cell varies along with the increasing applied current densities. Elemental carbon balance marked includes the unreacted CO_2_, CO produced and the CO_2_ loss into the solution phase. Experimental conditions include a 0.1 M KHCO_3_ electrolyte with a circulating flow rate of 8.2 mL/min and an inlet CO_2_ flow rate of 58 mL/min under ambient conditions (∼25 °C ± 2).

Therefore, on this basis, it is hypothesized that the effluent gas volume may either stay the same or decrease based on the reactions above. Following bicarbonate formation (Reaction 19), a decrease in effluent gas volume is expected due to the conversion of 2 mol of CO_2(g)_ dissolved in the solution into 1 mol of H_2(g)_. Alternatively, if the reaction path aligns with carbonate formation (as per Reaction 20), the effluent gas volume remains unchanged, given that each mole of CO_2(g)_ absorbed into the solution is precisely compensated by the generation of a mole of H_2(g)_. With both reactions occurring simultaneously, the combined effect leads to a net decrease in gas volume and falls within the shaded area between the blue and red dashed lines in Figure 6a.

Contrary to the initial hypothesis, Figure 6a reveals a linear increase in the volume of effluent gas observed throughout the experiment. This suggests that H_2_ production significantly surpasses CO_2_ consumption in the HER region, which is in line with the subsequent analysis of effluent gas molar flow rate, as depicted in Figure 6b. The molar flow rate of the H_2_ production increases linearly across current densities from 450 to 600 mA/cm^2^, surpassing the steady rate of CO_2_ loss in the solution. This discrepancy between the H_2_ generation and CO_2_ consumption accounts for the unexpected increase in effluent gas volume flow rate.

Additionally, a similar analysis of Faraday efficiency for H_2_ and CO production is conducted for the HER region, as is performed for the ECO_2_RR region. Figure S5 demonstrates that the total Faraday efficiency exceeds 98%, highlighting the high selectivity of these reactions and effectively ruling out the possibility of other electrochemical reductions occurring.

Consequently, the observed pattern of CO_2_ loss in the HER region also results from a nonelectrochemical process but is different from the phenomenon observed within the ECO_2_RR region.

**Figure 7:**
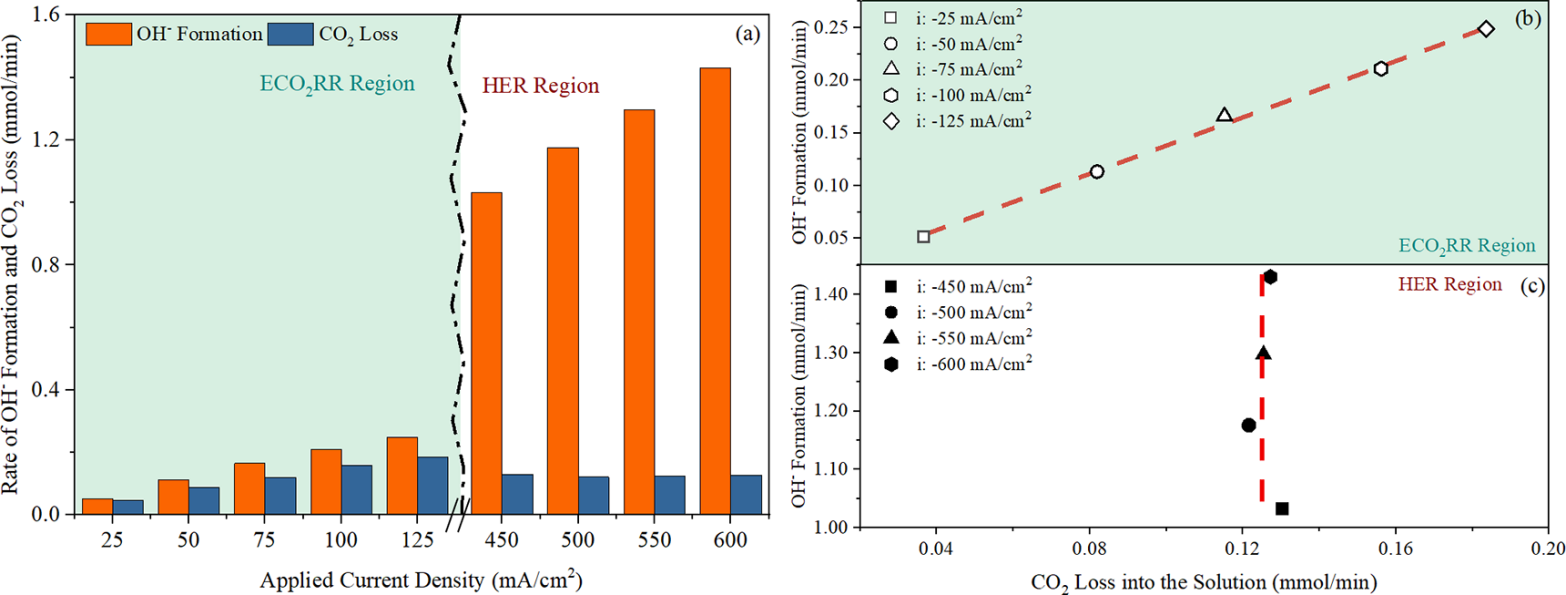
(a) Demonstrates the molar rate of CO_2_ loss into the solution phase alongside OH^–^ production, as inferred from the quantification of CO and H_2_ generation, plotted against the applied current density. ECO_2_RR region indicates that CO generation dominates while H_2_ production prevails in the HER region. (b,c) presents the correlation between the CO_2_ loss into the solution phase and the OH^–^ formation rate, in the ECO_2_RR and HER region, respectively, data derived from (a). Experimental conditions include a 0.1 M KHCO_3_ electrolyte with a circulating flow rate of 8.2 mL/min and a constant inlet CO_2_ flow rate of 58 mL/min under ambient conditions (∼25 °C ± 2).

Comparative analysis has revealed significant differences in detail between the mechanisms of CO_2_ loss in the solution phase under the HER and the ECO_2_RR conditions, demonstrated in Figure 7.

Within the ECO_2_RR domain, as shown in Figure 7a, increasing current densities leads to a linear increase in the OH^–^ production rate, which is accompanied by an increase in CO_2_ loss. This linear correlation, depicted in Figure 7b, is in alignment with theoretical analyses in Section 2.2.1, supporting the hypothesis that the formation of (bi)carbonate ions is occurring. In contrast, the HER domain presents a puzzle: Although a linear increase in OH^–^ generation occurs as expected along with increasing the applied current density, a bottleneck is observed on the CO_2_ loss into the solution phase. Figure 7c shows that the rate of CO_2_ loss remains unchanged at around 0.124 mmol/min. It implies that CO_2_ availability at the reaction sites does not increase proportionally to the OH^–^ production. This presents a fundamental limit to the formation of (bi)carbonate ions.

**Figure 8:**
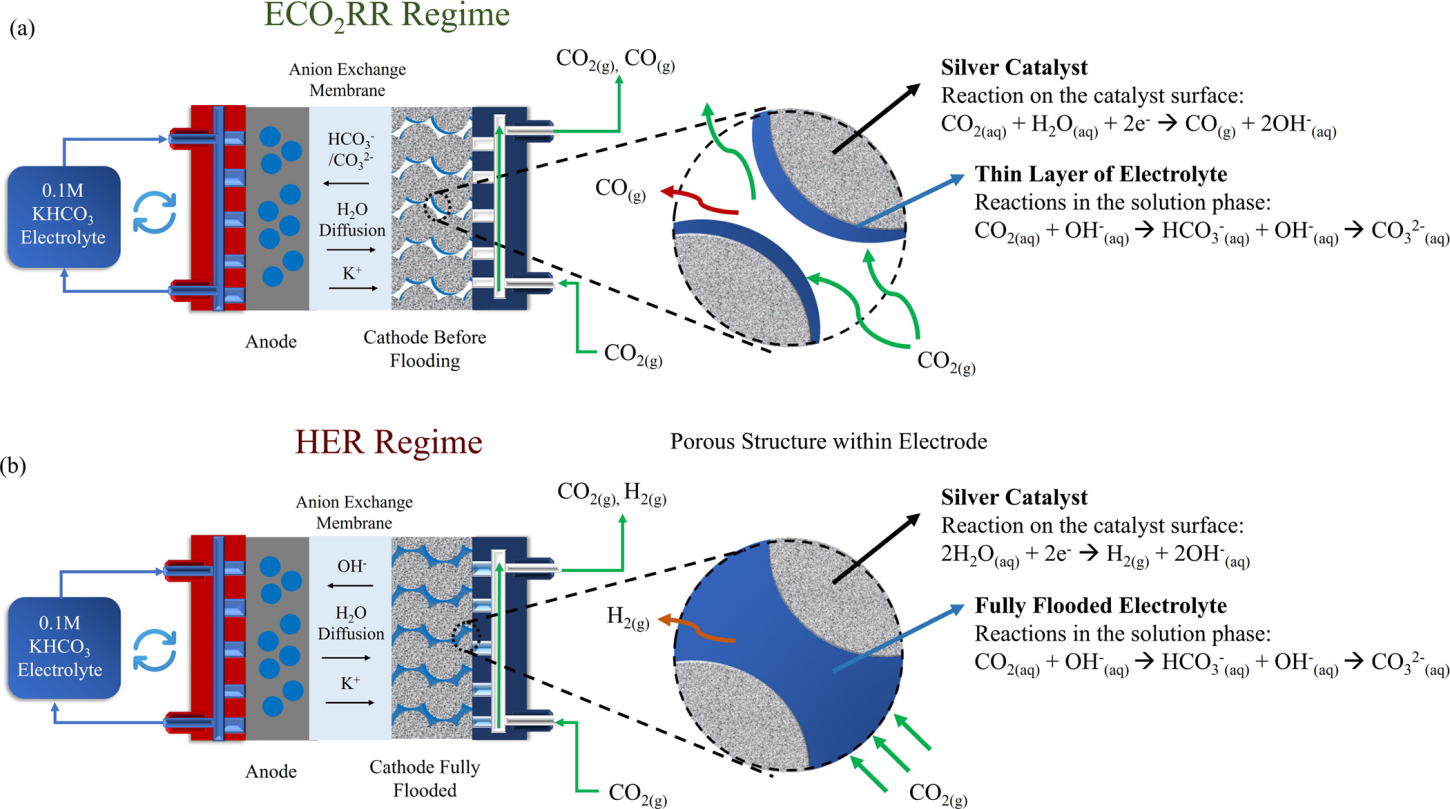
Diagram illustrates the change of the CO_2_ delivery from the ECO_2_RR regime to the HER regime due to the “flooding” issue. (a) Illustrate the sufficient CO_2_ delivery under the ECO_2_RR region, with a thin layer of electrolyte surrounding the electrode (b) showcasing the limited CO_2_ delivery under the HER region, where the porous cathode is fully flooded by the electrolyte. Cathode: 50 μm thickness-silver membrane with 0.2 μm size of the porous structure. Circulating electrolyte: 0.1 M KHCO_3_.

This phenomenon can be attributed to the ‘flooding’ issue at the cathode, as depicted in Figure 8a,b. While the exact mechanism triggering flooding remains ongoing research, existing literature suggests that it may begin with a decrease in capillary pressure between the electrolyte and the gas diffusion electrode’s porous structure, a process driven by the electrochemical potential.^20,40,41^ When the electrolyte, introduced from the anode, begins to occupy a significant portion of the pores within the gas diffusion electrode. This process extends the diffusion distance of CO_2_ to the catalyst’s active sites in the liquid, thereby impeding CO_2_ accessibility to the reaction sites.^7,42^ Such electrolyte accumulation eventually saturates the gas diffusion layer, leading to a complete blockage of the channels for gas-phase CO_2_ delivery. Consequently, cathode flooding facilitates a shift from ECO_2_RR to HER as water is still sufficiently supplied, fundamentally reducing the CO_2_ electrolysis system’s efficiency.^20−22^ Experimental evidence reinforces the theory of cathode flooding; the minor flooding of the electrolyte into the gas outlet was observed consistently during the HER region tests, suggesting that the cathode is flooded.

Figure 8a illustrates that in the ECO_2_RR region, prior to flooding, the electrolyte forms only a thin layer at the cathode interface. Here, ECO_2_RR mainly occurs at the solid–liquid double-phase boundary, extending 10–1000 nm into the liquid phase.^7^ In this scenario, adsorbed CO_2_ accumulates at the top of the liquid surface around 1 nm, reaching densities approximately 20 times higher than that found in bulk liquid water,^7^ facilitating efficient CO_2_ reduction and (bi)carbonate ions formation. However, as the HER becomes the dominant reaction, depicted in Figure 8b, the cathode becomes fully immersed in the liquid, resulting in a bulk environment where the gas diffusion layer’s capacity to transport CO_2_ is severely compromised. Consequently, only a limited amount of CO_2_ dissolves in the liquid for reaction with OH^–^, marking a significant shift from efficient gas-phase CO_2_ delivery to a scenario where CO_2_ mass transport is drastically restricted by flooding. This transition from a preflooding to a fully flooded cathode state is consistent with the distinct CO_2_ loss patterns observed in the ECO_2_RR and HER domains, as shown in Figure 7b,c. Specifically, CO_2_ loss rates exhibit a linear increase up to 0.18 mmol/min in the ECO_2_RR domain, indicative of adequate CO_2_ delivery. Additionally, testing higher inlet CO_2_ flow rates revealed no significant increase in CO yield, as depicted in Figure S6, suggesting saturation of CO_2_ entering the system. In contrast, the HER domain shows a stable loss rate at 0.12 mmol/min, with the amount of CO_2_ absorbed in the electrolyte remaining constant despite increased electrical input. This indicates that flooding leads to a bottleneck in CO_2_ mass transport, severely limiting the efficiency of CO_2_ reduction and the (bi)carbonate ions generation as the cathode becomes fully submerged in the aqueous electrolyte.

Although the experiments in this work have explored CO_2_ loss into the solution phase, suggesting (bi)carbonate formation within the ECO_2_RR domain and the emergence of the ‘flooding’ phenomenon as HER predominates, this still does not fully capture the complex carbon dynamics throughout the cell. Particularly, the CO_2_ loss investigation has been confined to the cathodic compartment. This limitation highlights the need to expand CO_2_ loss studies beyond the cathode, aiming to thoroughly investigate cross-membrane (bi)carbonate migration, which could offer a more holistic view of the process. Previous studies^36,37^ collected and determined the gaseous products effluent from the anode, where the ratio of CO_2_ versus O_2_ is accessed as a key indicator for determining the main ion species (OH^–^/HCO_3_^–^/CO_3_^2–^) traveling through the membrane. This work adopts a straightforward method to link the cathodic and anodic domains, monitoring pH changes in the electrolyte under both ECO_2_RR and HER conditions, and during and after the electrolysis. This dual-phase observation offers insights into ion species migration across the membrane, potentially bridging gaps in current understanding and enhancing the detailed study of these processes.

### 3.3 pH Changes in Electrolytes

**Figure 9:**
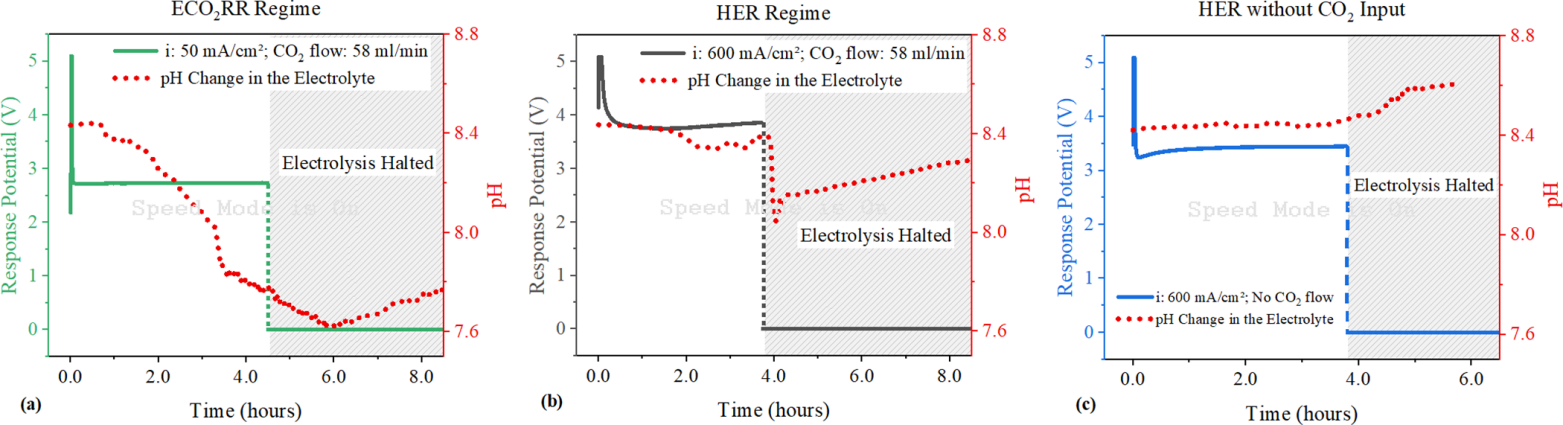
Chronopotentiometry test outcomes; (a) at 50 mA/cm^2^ under the ECO_2_RR region, CO_2_ supplied at 58 mL/min, including pH observations during and post-test; (b) at 600 mA/cm^2^ under the HER region, CO_2_ supplied at 58 mL/min, with continuous pH tracking during and post-test; (c) at 600 mA/cm^2^ without CO_2_, monitoring electrolyte pH during and post-test. Experimental conditions include a 0.1 M KHCO_3_ electrolyte with a circulating flow rate of 8.2 mL/min under ambient conditions (∼25 °C ± 2). Fresh batches of the electrolyte were prepared for each experiment.

Analysis from previous sections indicates that in the ECO_2_RR region, (bi)carbonate ions are the primary species migrating across the membrane into the electrolyte, driven by the applied electrical field. Reactions 7 and 8 suggest that this migration eventually releases CO_2_ into the electrolyte, saturating the 0.1 M KHCO_3_ solution, resulting in a pH decrease.^34,36,43^ This hypothesis is validated by the experiment results, shown in Figure 9a. In a chronopotentiometry test with an applied current density of 50 mA/cm^2^ under a CO_2_-rich atmosphere for 4 h, the electrolyte’s pH drops from 8.4 to 7.8, and further to 7.6 when the current is turned off (with CO identified as the primary product in effluent gas, as confirmed by gas chromatograph, shown in Figure S7a). The initial decrease in pH results from the migration and subsequent neutralization of (bi)carbonate ions, which form CO_2_ in the electrolyte. The further pH reduction, after the current has been switched off, stems from the diffusion of residual (bi)carbonate ions from the cathode, triggering CO_2_ released at the anode. This suggests that (bi)carbonate ion production exceeds their migration rate through the membrane, leading to accumulation at the cathode. Consequently, once the concentration of (bi)carbonate ions surpasses the critical solubility threshold, precipitation of (bi)carbonate salts can occur within the porous structure of the cathode.

Conversely, within the HER domain, the production of hydroxide ions significantly exceeds the formation of (bi)carbonate ions due to the “flooding” phenomenon detailed in Section 3.2. Thus, hydroxide ions become the dominant species migrating through the membrane, owing to their higher production rates and closer proximity to the membrane relative to (bi)carbonate ions. A chronopotentiometry test is conducted at an applied current density of 600 mA/cm^2^ in a CO_2_ atmosphere, with pH monitored during and after the test (Figure S7b shows effluent gas composition, confirming the HER dominance). Results in Figure 9b indicate pH remains nearly constant at 8.4 throughout the test, then drops sharply to 8.0 when the electricity is turned off.

The initial change in pH supports the theory that OH^–^ ions move across the membrane and react with protons at the anode, without affecting the overall pH of the electrolyte. This theory is confirmed by a controlling experiment shown in Figure 9c, where chronopotentiometry is performed at the same current density in the absence of CO_2_. It demonstrates a pure water-splitting scenario, where the movement of OH^–^ ions alone across the membrane and their subsequent neutralization in the electrolyte do not change the pH, consistent with the behavior observed in the HER domain. In the presence of CO_2_, after turning off the electricity, the sudden drop in pH suggests that (bi)carbonate ions accumulated at the cathode are diffusing through the membrane and reaching the electrolyte.

The changes in pH within the electrolyte, coupled with the previous investigation of the CO_2_ loss into the solution, raise a significant concern regarding the primary cause of (bi)carbonate salt precipitation, suggesting that the HER might be the main contributor. This observation seems to conflict with the conclusions drawn in Section 3.2, where the ″flooding″ associated with HER was thought to decrease the formation rate of (bi)carbonate ions, thereby reducing the possibility of salt precipitation. It is crucial to understand, however, that the precipitation of salts is not directly linked to the formation rate of (bi)carbonate ions but rather to the balance of their creation at the cathode and their migration rates across the membrane, as this balance affects the accumulation rate of the (bi)carbonate at the cathode.

As shown in Figure 7b,c, at an applied current density of 50 mA/cm^2^ (ECO_2_RR region), CO_2_ loss due to (bi)carbonate formation is 0.09 mmol/min, which is less than the 0.12 mmol/min generation rate at 600 mA/cm^2^ (HER region). Also, in the HER domain, as indicated by pH changes in Figure 9b, the (bi)carbonate ions’ migration rate is nearly zero because OH^–^ ions predominate. Consequently, a high generation rate coupled with a negligible migration rate of the (bi)carbonate ions will speed up the salt accumulation, increasing the likelihood of salt precipitation at the cathode within the HER region. This outcome is corroborated by experimental observations, where the inlet channel for CO_2_ within the cell is found to be completely blocked after 7 h of operation, as depicted in Figure S8.

While pH changes in the electrolyte can serve as an indirect marker for ion species migration through the membrane, a comprehensive understanding of salt precipitation and accurate quantification of salt accumulation at the gas diffusion electrode demands an in-depth analysis of carbon flow within the cell, including the transition phase from ECO_2_RR to HER. Utilizing sophisticated methods like attenuated total reflection surface-enhanced infrared absorption spectroscopy (ATR-SEIRA), micro area Raman spectroscopy, and EDX/ICP–MS can uncover intricate changes from ECO_2_RR to HER transition and the evolution from preflooding to fully flooded states in the gas diffusion electrode, offering deeper mechanistic insights. This field represents a valuable avenue for future investigations.

## 4 Conclusion

This study examines critical operational issues in an MEA-type CO_2_ electrolyzer utilizing a pure silver cathode, with a particular focus on CO_2_ loss through the formation of (bi)carbonate (HCO_3_^–^/CO_3_^2–^) ions in the solution phase. Molar concentrations of effluent gases (CO_2_, CO, and H_2_) are systematically monitored relative to applied current densities. The results indicate that CO production predominates at current densities below 125 mA/cm^2^, defining the ECO_2_RR region, while a significant shift toward H_2_ generation occurs at higher currents above 450 mA/cm^2^, marking the HER region.

Notably, a 7.7% reduction in the gas volume flow rate exiting from the cell is observed in the ECO_2_RR region compared to the baseline inlet CO_2_ flow rate. Faradaic efficiencies, consistently high at 95–99% for the ECO_2_RR region and 97–98% for the HER region, suggest the absence of significant alternative electrochemical reactions influencing this reduced flow rate. Further analysis confirms that interactions between CO_2_ and OH^–^ ions at lower current densities contribute to (bi)carbonate ion formation, where CO_2_ loss is demonstrated to be proportional to the current density, reaching up to 0.18 mmol/min. These processes reduce both the volume flow rate and CO_2_ utilization efficiency, significantly impacting system performance. Monitoring of pH changes in the electrolyte reveals a decrease from 8.4 to 7.8, which further declines to 7.6 once the current is removed. This initial decrease aligns with the migration and subsequent neutralization of (bi)carbonate ions, while the further reduction in pH after the removal of current suggests their diffusive movement away from the cathode. This indicates that the production rate of (bi)carbonate ions exceeds their migration rate across the membrane, potentially leading to a buildup of potassium (bi)carbonate salt at the cathode and reducing the lifespan of the cells. However, to precisely quantify each source of CO_2_ loss to solution phase—specifically, the loss from potassium (bi)carbonate salt precipitation at the cathode and the CO_2_ released from the anolyte—further development of the current measurement system is required. This enhanced system should enable the separation of the liquid and gaseous phases from the anolyte and facilitate tracking of carbon in both phases to accurately determine the extent of carbon migration through the membrane.

At higher current densities of the HER region, CO_2_ loss is constant at approximately 0.12 mmol/min. This consistent CO_2_ loss suggests an alternative mechanism likely due to limitations in CO_2_ transport to the cathode, consistent with the flooding phenomena due to water transport across the anion exchange membrane. The pH remains at 8.4 across all current densities, indicative of dominant OH^–^ migration across the membrane, then drops to 8.0 once the current is removed, indicating the diffusion of accumulated (bi)carbonate ions from the cathode through the membrane postformation. The dominant migration of OH^–^ ions across the membrane hinders the movement of (bi)carbonate ions formed at the cathode and further exacerbates the accumulation of these salts, thereby degrading cell performance.

Addressing these challenges to improve CO_2_ utilization efficiency requires innovative approaches to limit the generation of HCO_3_^–^/CO_3_^2–^ ions, a task complicated by the rapid nature of the CO_2_–OH^–^ neutralization process. Potential strategies include enhancing the dispersion of OH^–^ ions to minimize their interaction with incoming CO_2_ and developing cost-effective methods for the capture and recycling of CO_2_ released at the anode. Furthermore, improving water management at the cathode and increasing the migration rate of (bi)carbonate ions across the membrane may reduce salt precipitation and extend the life of the cells. With insights into the formation mechanisms of (bi)carbonate ions and salt accumulation at the cathode, this study provides valuable knowledge for optimizing MEA-type CO_2_ electrolyzers.

## Supporting Information Available

SEM images of electrodes employed in the experiments, chronopotentiometry test, and reproducibility and reliability tests conducted at two different CO_2_ flow rates, Faraday efficiency measurements in the ECO_2_RR region and HER region, the mole concentration of effluent gaseous products at the applied current density 50 and 600 mA/cm^2^, respectively, also a descriptive experiment for the cell blockage test (PDF)
