# Supplementary material for: CO2 Loss into Solution: An Experimental Investigation of CO2 Electrolysis with a Membrane Electrode Assembly Cell
Source: ACS Appl Energy Mater. 2024 Sep 5;7(18):7712–23. doi: 10.1021/acsaem.4c01101 (PMC11423278; doi:10.1021/acsaem.4c01101)
Supplement: Supplementary file 1 — ae4c01101_si_001.pdf [file ae4c01101_si_001.pdf]

Supporting Information

CO_2 Loss into Solution: An Experimental Investigation of CO_2 Electrolysis with a Membrane Electrode Assembly Cell

Weiming Liu[1*], Harry Dunne[1], Bernardo Ballotta[1], Allyssa A Massie[1], Mohammad Reza. Ghaani[2]*, Kim McKelvey[3], Stephen Dooley[1]*

[1] School of Physics, [2] School of Engineering, Department of Civil, Structural & Environmental Engineering, Trinity College Dublin, Dublin, Ireland.

[3]MacDiarmid Institute for Advanced Materials and Nanotechnology, School of Chemical and Physical Sciences, Victoria University of Wellington, Wellington, New Zealand.

**Corresponding author: Weimingliu@tcd.ie, mohammad.ghaani@tcd.ie, stephen.dooley@tcd.ie.*

Table of Contents

FIGURE S1. SCANNING ELECTRON MICROSCOPY (SEM) DEPICTION OF THE CATHODE. PANEL (A) ILLUSTRATES THE CATHODE'S FRONT SURFACE, ORIENTED TOWARDS THE MEMBRANE, WHILE PANEL (B) DISPLAYS THE BACK SURFACE, FACING THE FLOW CHANNEL. S-2

FIGURE S2. SCANNING ELECTRON MICROSCOPY (SEM) DEPICTION OF THE ANODE. PANEL (A) ILLUSTRATES THE ANODE'S FRONT SURFACE, COMPOSED OF IRO_2, WHICH IS ORIENTED TOWARDS THE MEMBRANE. IN CONTRAST, PANEL (B) DISPLAYS THE BACK SURFACE, FEATURING THE CARBON FIBRE SUBSTRATE, FACING THE FLOW CHANNEL. S-2

FIGURE S3. CO PRODUCTION AS A FUNCTION OF APPLIED CURRENT DENSITY WITH TWO SETS OF INLET FLOW RATES OF CO_2, 58 ML/MIN AND 145 ML/MIN. S-3

FIGURE S4. CHRONOPOTENTIOMETRY MEASUREMENT FOR CO_2 REDUCTION IS ILLUSTRATED AS A FUNCTION OF RESPONSE POTENTIAL AGAINST APPLIED CURRENT DENSITY RANGING FROM -25 MA/CM^2 TO -600 MA/CM^2. CONDITIONS: 0.1 M $KHCO_3$ ELECTROLYTE, CIRCULATING FLOW RATE OF 8.2 ML/MIN, CO_2 AT A FLOW RATE OF 58 ML/MIN, AT AMBIENT CONDITIONS (~25°C ± 2). S-4

FIGURE S5. FARADAY EFFICIENCY (%) OF CO AND H_2 GAS PRODUCTS GENERATED AT THE CATHODE SIDE AS A FUNCTION OF THE APPLIED CURRENT DENSITY. CONDITIONS: 0.1 M $KHCO_3$ ELECTROLYTE, CIRCULATING FLOW RATE OF 8.2 ML/MIN, CO_2 AT A FLOW RATE OF 58 ML/MIN, AT AMBIENT CONDITIONS (~25°C ± 2)... S-5

FIGURE S6. CO YIELD IN MOLAR PER MINUTE AGAINST APPLIED CURRENT DENSITY WITH TWO SETS OF INLET CO_2, 58 ML/MIN AND 145 ML/MIN. CONDITIONS: 0.1 M KHCO3 ELECTROLYTE, CIRCULATING FLOW RATE OF 8.2 ML/MIN, CO_2 AT A FLOW RATE OF 58 ML/MIN, AT AMBIENT CONDITIONS (~25°C ± 2). S-6

FIGURE S7. THE GASEOUS PRODUCT CONCENTRATION IN PERCENTAGE AS A FUNCTION OF APPLIED CURRENT DENSITY (A) AT -50 MA/CM2, AND (B) AT -600 MA/CM2. CONDITIONS: 0.1 M KHCO3 ELECTROLYTE, CIRCULATING FLOW RATE OF 8.2 ML/MIN, CO2 AT A FLOW RATE OF 58 ML/MIN, AT AMBIENT CONDITIONS (~25°C ± 2). S-7

FIGURE S8. THE FLOW RATE OF THE EFFLUENT GAS PRODUCTS FROM THE CATHODE OUTLET AS A FUNCTION OF TIME OVER CYCLIC VOLTAMMETRY MEASUREMENT. CONDITIONS: 0.1 M $KHCO_3$ ELECTROLYTE, CIRCULATING FLOW RATE OF 8.2 ML/MIN, CO_2 AT A FLOW RATE OF 10 ML/MIN, AT AMBIENT CONDITIONS (~25°C ± 2). S-7

1. Scanning Electron Microscopy for Cathode and Anode

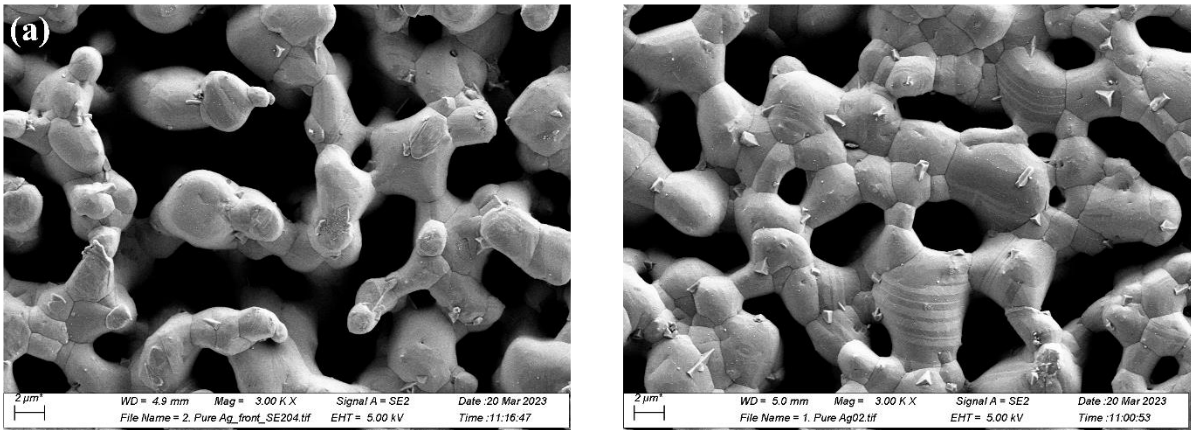

Figure S1. Scanning Electron Microscopy (SEM) Depiction of the Cathode. Panel (a) illustrates the cathode's front surface, oriented towards the membrane, while Panel (b) displays the back surface, facing the flow channel.

This figure presents a cathode composed of pure metallic silver, characterized by a porous membrane with an average pore size of 0.2 micrometres.

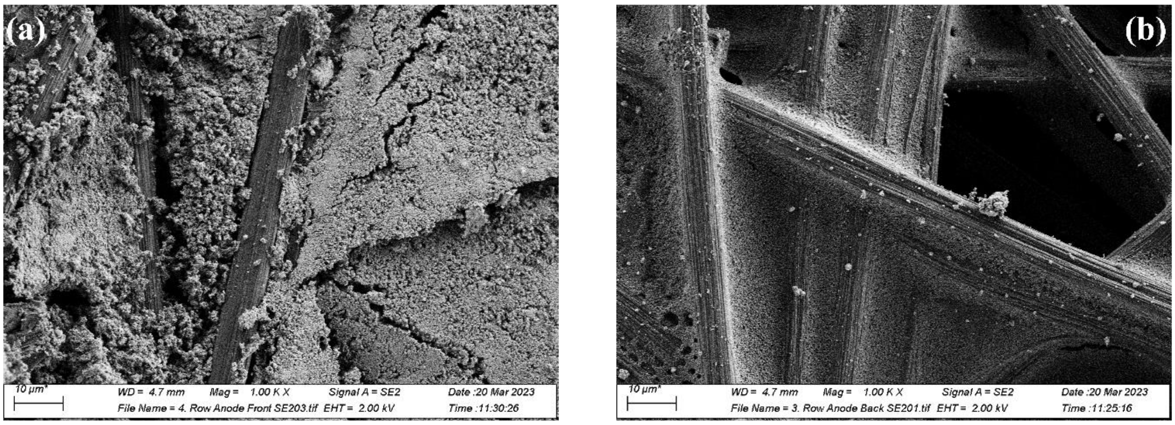

Figure S2. Scanning Electron Microscopy (SEM) Depiction of the Anode. Panel (a) illustrates the anode's front surface, composed of IrO_2, which is oriented towards the membrane. In contrast, Panel (b) displays the back surface, featuring the carbon fibre substrate, facing the flow channel.

This figure showcases an anode made of an IrO_2/carbon fibre composite, offering a detailed view of its microstructure.

2. Reproducibility and Reliability Test

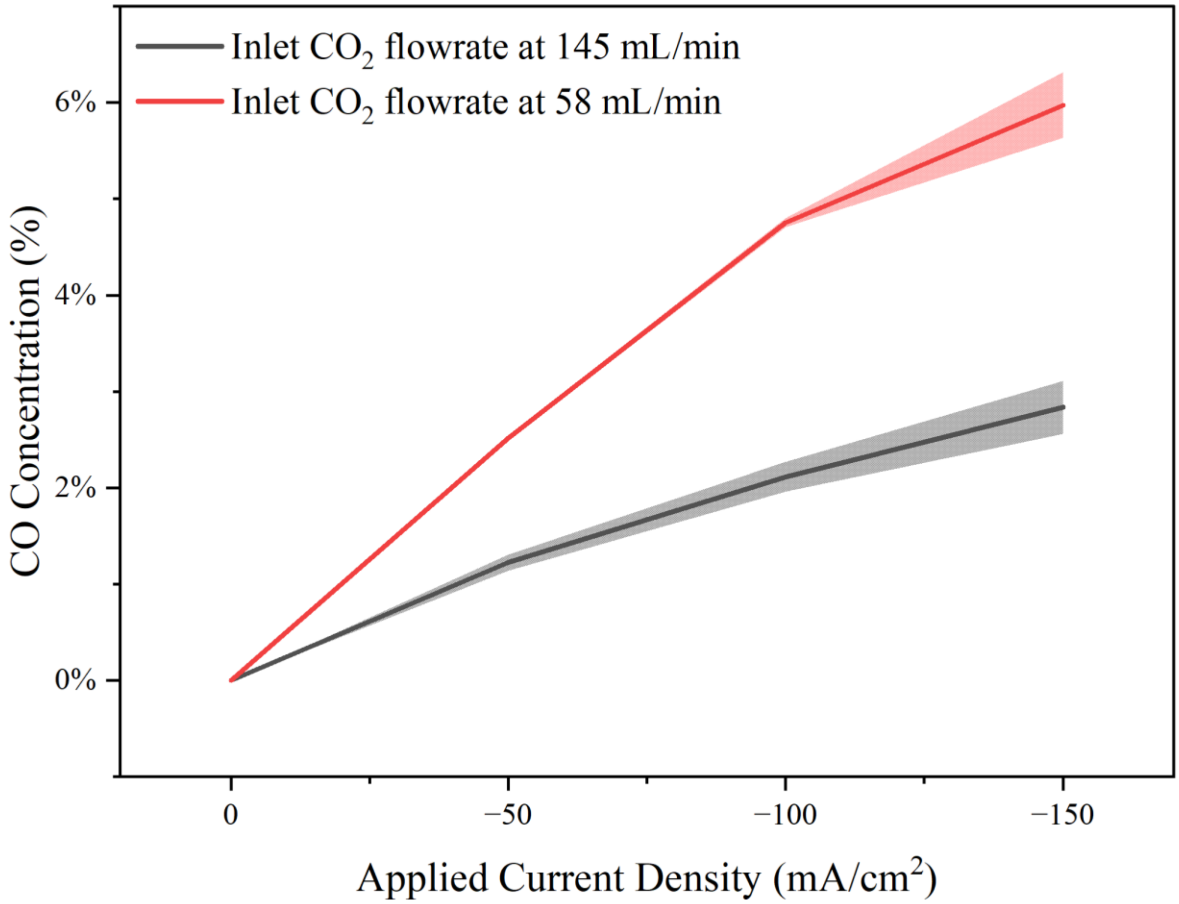

Figure S3. CO mole concentration as a function of applied current density with two sets of inlet flow rates of CO_2, 58 mL/min and 145 mL/min.

This graph illustrates the mole percentage of CO produced, as determined by gas chromatography, under two different CO_2 flow rates. The analysis includes data from three distinct fabrications of cells at each of the two CO_2 flow rates, 58 mL/min and 145 mL/min, showcasing the uniformity in CO production across different cell constructions. Both flow rates exhibited a standard deviation below 0.03 and a coefficient of variance less than 0.1, confirming high reproducibility. At an applied current density above 150 mA/cm^2, the response potential during Chronopotentiometry measurements begins to oscillate (see Supporting Information, Figure S4), suggesting the onset of the flooding issue, which compromises the utility of products from the cathode for evaluating reproducibility and repeatability of cell assemblies and experimental procedures.

3. Chronopotentiometry Test

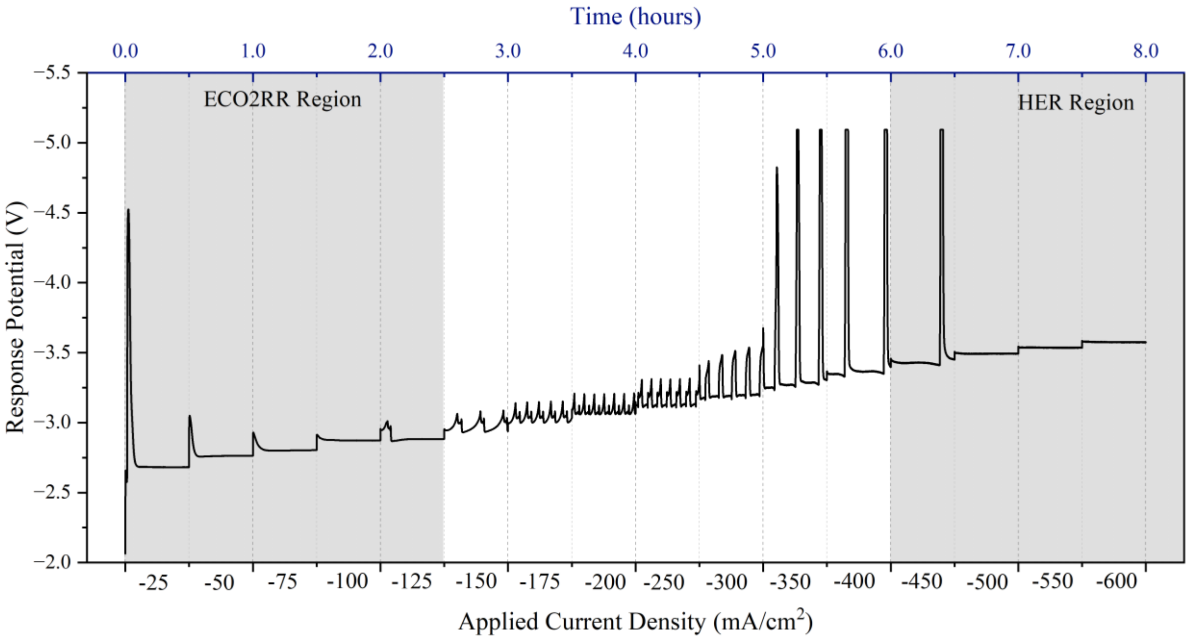

Figure S4. Chronopotentiometry measurement for CO_2 reduction is illustrated as a function of response potential against applied current density ranging from 25 mA/cm^2 to 600 mA/cm^2. Conditions: 0.1 M $KHCO_3$ electrolyte, circulating flow rate of 8.2 mL/min, CO_2 at a flow rate of 58 mL/min, at ambient conditions (~25°C ± 2).

In this figure, the ECO2RR region has been marked in grey at the applied current density below 125 mA/cm^2 with CO production dominating and the HER region is highlighted in grey at the applied current density above 450 mA/cm^2. The region in between presents oscillation, indicating the flooding on the electrode.

4. Faraday Efficiency Measurement

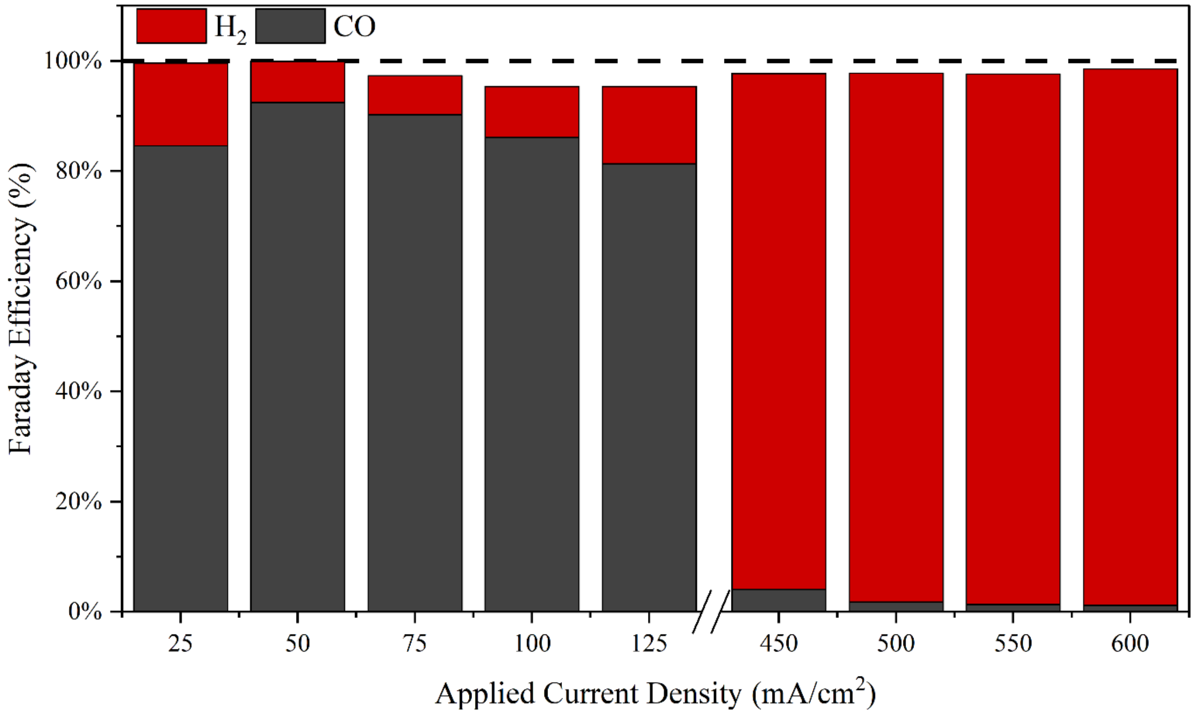

Figure S5. Faraday efficiency (%) of CO and H_2 gas products generated at the cathode side as a function of the applied current density. Conditions: 0.1 M $KHCO_3$ electrolyte, circulating flow rate of 8.2 mL/min, CO_2 at a flow rate of 58 mL/min, at ambient conditions (~25°C ± 2).

At the applied current density below 125 mA/cm^2, the CO production is dominating, refer to the ECO2RR region and at the applied current density above -450 mA/cm^2, the H_2 production prevails, denoted as HER region. The total Faraday Efficiency of CO and H_2 is nearly 100%, which suggests there are no other products resulting from the electrochemical reduction.

5. Two Levels of Inlet CO_2 Flow Rate Test

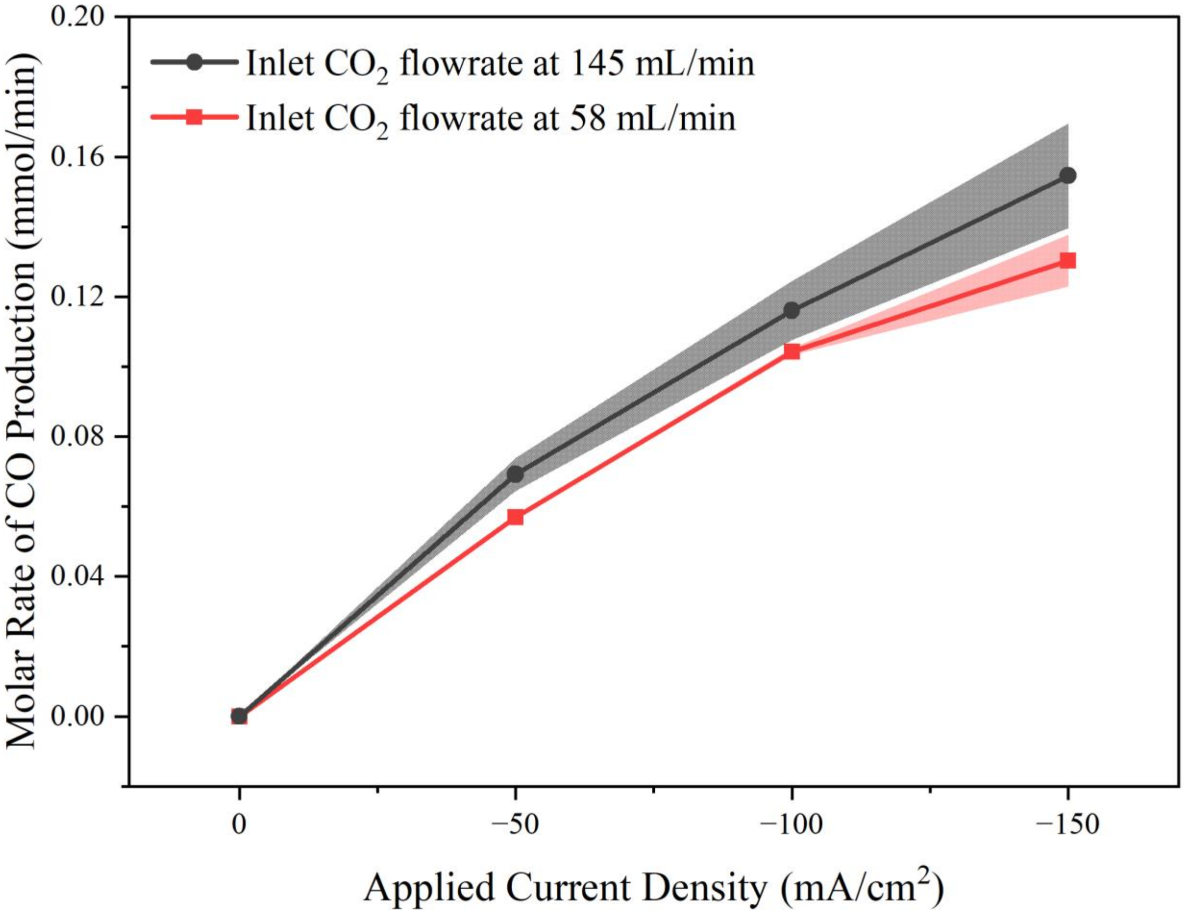

Figure S6. CO yield in molar per minute against applied current density with two sets of inlet CO_2, 58 mL/min and 145 mL/min. Conditions: 0.1 M $KHCO_3$ electrolyte, circulating flow rate of 8.2 mL/min, CO_2 at a flow rate of 58 mL/min, at ambient conditions (~25°C ± 2).

The graph elucidates that although increasing the CO_2 flow rate to 145 mL/min leads to a slight uptick in CO production, the differential is minimal. This observation suggests a saturation status for inlet CO_2 with a flow rate of 58 mL/min within the system, where increasing the CO_2 flow rate does not proportionately enhance CO production.

6. Gas Concentration and pH Monitoring

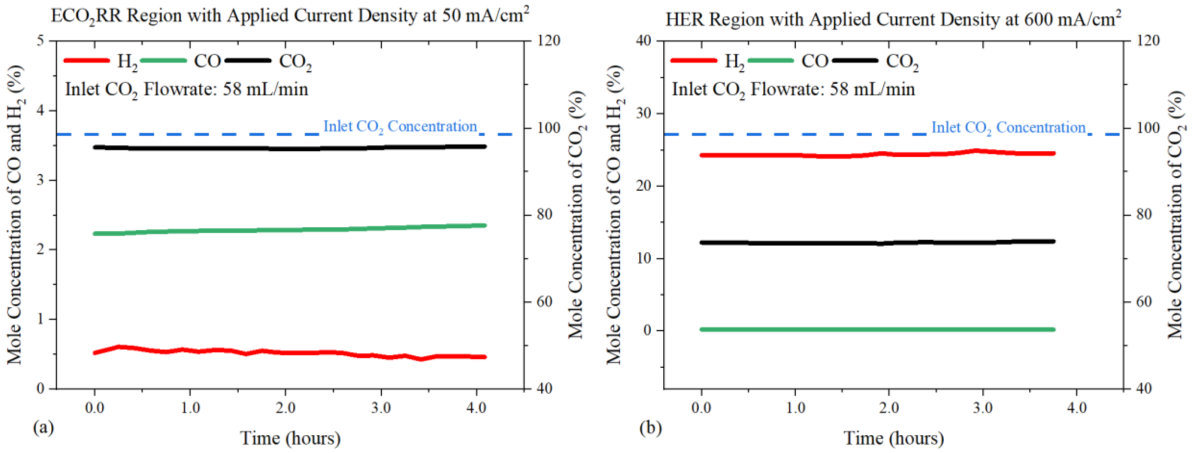

Figure S7. The gaseous product mole concentration in percentage as a function of applied current density (a) at 50 mA/cm^2, and (b) at 600 mA/cm^2. Conditions: 0.1 M $KHCO_3$ electrolyte, circulating flow rate of 8.2 mL/min, CO_2 at a flow rate of 58 mL/min, at ambient conditions (~25°C ± 2).

This figure presents the concentration of gases produced, as determined by gas chromatography, across varying current densities. (a) At an applied current density of 50 mA/cm², there's a noticeable predominance of CO production, highlighting the ECO2RR region. (b) Conversely, at 600 mA/cm², H_2 generation significantly dominates other reactions, indicating the onset of the hydrogen evolution reaction (HER).

7. Blockage on the Cell Inlet

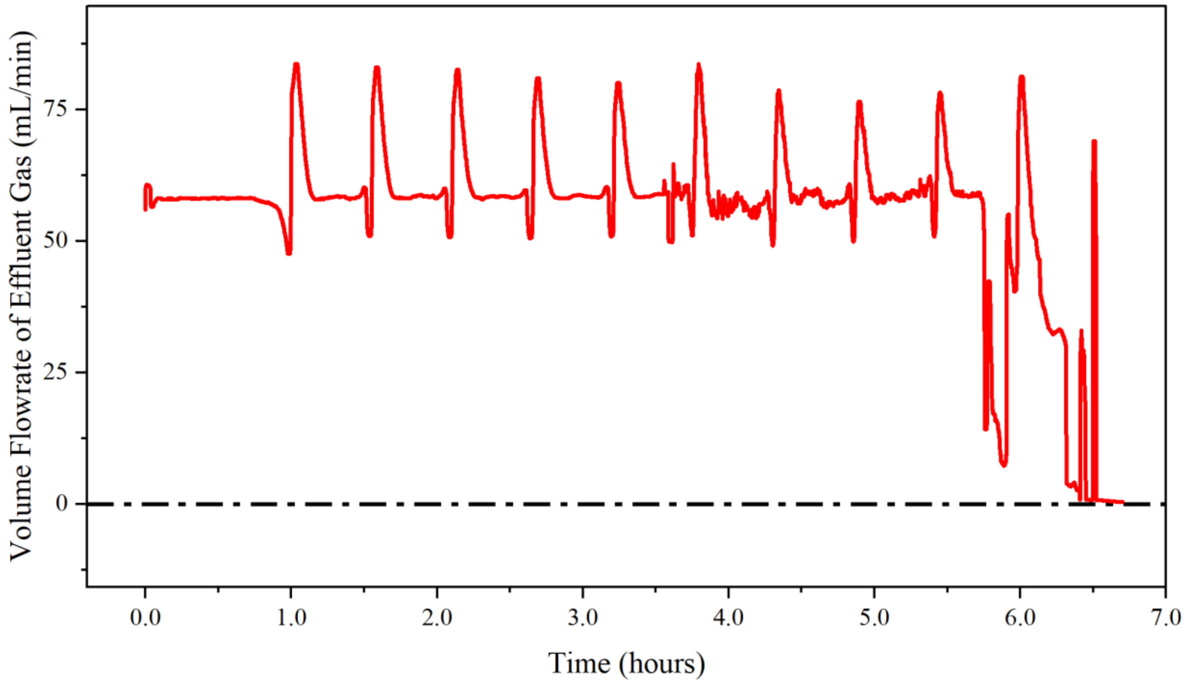

Figure S8. The flow rate of the effluent gas products from the cathode outlet as a function of time over Cyclic Voltammetry measurement. Conditions: 0.1 M $KHCO_3$ electrolyte, circulating flow rate of 8.2 mL/min, CO_2 at a flow rate of 10 mL/min, at ambient conditions (~25°C ± 2).

The flow rate went down to almost 0 when it came to 5.5 hours (cycle #10). It indicated the blockage of the cell's inlet, which is in line with our hypothesis that salt precipitation will block the cell channel.
